# Effects of catheter-based extradural collagenase chemonucleolysis on pain scores and lumbar function in patients with lumbar disc herniation

**DOI:** 10.3389/fmed.2026.1807178

**Published:** 2026-04-13

**Authors:** Yongfu Feng, Yuanxin Huang, Jie Yuan, Guoping Song, Guanfeng Shang, Qian Zhang, Xiaoyun Luo, Yun Xu

**Affiliations:** 1 Department of Pain, The Second Affiliated Hospital of Guizhou Medical University, Kaili, Guizhou, China; 2 Department of Anesthesiology, Affiliated Hospital of Guizhou Medical University, Guiyang, Guizhou, China; 3 Affiliated Hospital of Zunyi Medical University, Zunyi Medical University, Zunyi, Guizhou, China; 4 Department of Pain, Guiyang Fourth People's Hospital, Guiyang, Guizhou, China; 5 Medical Imaging Specialty, The Second Affiliated Hospital of Guizhou Medical University, Kaili, Guizhou, China; 6 Department of Rehabilitation, Tianzhu County People's Hospital, Tianzhu, Guizhou, China

**Keywords:** catheter, extradural collagenase chemonucleolysis, lumbar disc herniation, lumbar function, pain

## Abstract

**Aim:**

Lumbar disc herniation (LDH) is a leading cause of radicular pain and disability. While conventional intradiscal collagenase chemonucleolysis (ICCN) is a minimally invasive alternative to surgery, it is limited by suboptimal drug targeting and recurrence. Catheter-based extradural collagenase chemonucleolysis (CECCN) was developed to deliver the enzyme directly to the herniated fragment. This research aimed to compare the clinical efficacy and safety of CECCN versus ICCN.

**Methods:**

A total of 300 patients with single-level, symptomatic lumbar disc herniation refractory to conservative treatment were included. The CECCN group (*n* = 150) consisted of patients prospectively enrolled between January 2025 and December 2025. Data for this group were provided by five centers: the Department of Pain, The Second Affiliated Hospital of Guizhou Medical University (*n* = 70), the Department of Pain, Affiliated Hospital of Guizhou Medical University (*n* = 20), the Department of Pain, Affiliated Hospital of Zunyi Medical University (*n* = 20), the Department of Pain, Guiyang Fourth People’s Hospital (*n* = 20), and the Department of Rehabilitation, Tianzhu County People’s Hospital (*n* = 20). The ICCN group (*n* = 150) was established through retrospective review of medical records of patients who underwent conventional ICCN at the Department of Pain, The Second Affiliated Hospital of Guizhou Medical University between January 2023 and December 2024, met the same inclusion and exclusion criteria, and completed the predefined outcome assessments at the required follow-up time points. The outcomes were operation time, hospital stay, pain severity, lumbar function, clinical efficacy, quality of life and complications.

**Results:**

Operation time was longer in the CECCN group (*p* < 0.01), but hospital stay was comparable (*p* > 0.05). The CECCN group demonstrated significantly lower NRS scores at 7 days, 1, 3, as well as 6 months (all *p* < 0.05). At 3 and 6 months, the CECCN group also showed superior JOA scores (*p* < 0.05), lower ODI scores (p < 0.05), a higher excellent/good rate per Macnab criteria (*p* < 0.05), as well as better SF-36 scores (*p* < 0.05) relative to the ICCN group. The occurrence of complication rate presented significantly lower in the CECCN group (*p* < 0.05).

**Conclusion:**

CECCN is more effective than conventional ICCN for relieving pain, improving function and quality of life, and reducing complications in LDH patients. The superior outcomes support its role as an advanced percutaneous treatment option, despite a longer procedural time.

## Introduction

Lumbar disc herniation (LDH) belongs to a prevalent condition characterized by intervertebral disc degeneration. Under mechanical stress, the annulus fibrosus ruptures, allowing the nucleus pulposus to protrude and compress or irritate adjacent spinal nerves, resulting in lumbar and radicular symptoms (1). With accelerating societal pace and evolving work patterns, the incidence of LDH continues to rise, imposing a significant public health burden (2). Although many patients respond well to conservative treatments-such as physical therapy, analgesics, and epidural steroid injections-a subset continue to experience refractory pain, neurological deficits, and functional impairment (3, 4). For these individuals, surgical interventions like microdiscectomy have traditionally been the definitive option (5). However, surgery entails inherent risks, including infection, dural tears, epidural fibrosis, and spinal instability, alongside considerable economic costs and prolonged recovery (6).

Chemonucleolysis is a minimally invasive technique that involves percutaneous injection of hydrolytic enzymes into the intervertebral disc or epidural space to dissolve herniated nucleus pulposus, thereby alleviating nerve root compression (7). First described by Sussman in 1968, early procedures primarily used chymotrypsin. However, by the 1980s, increasing reports of systemic allergic reactions, spinal cord injuries, and other serious complications led to a decline in its use. Since the turn of the century, chymotrypsin-based chemonucleolysis has become rare, and research has largely shifted to collagenase-based protocols (8). Several clinical studies, as well as recent systematic and narrative reviews, have supported the efficacy and safety of intradiscal chemonucleolysis for LDH (8–10). Nevertheless, the conventional intradiscal collagenase chemonucleolysis (ICCN) approach presents several limitations: poor targeting of the herniated fragment, insufficient enzyme dwell time at the site of pathology, variability in enzymatic activity and dosing, and a notable recurrence rate attributable to persistent annular defects and residual inflammatory tissue (7, 8).

To overcome these drawbacks, catheter-based extradural collagenase chemonucleolysis (CECCN) has been developed as an advanced refinement of the technique. Unlike the intradiscal approach, which delivers collagenase centrally into the disc space, this method involves real-time fluoroscopically guided placement of a flexible microcatheter into the epidural space. The catheter is navigated—typically via a transforaminal or interlaminar route—to the immediate vicinity of the herniated material. This enables precise, localized delivery of collagenase directly onto the surface of the extrusion or sequestration. By maximizing contact between the enzyme and the pathological substrate while minimizing exposure to healthy disc and neural structures, the technique enhances therapeutic specificity. The catheter also allows controlled, low-pressure injection, promoting even distribution around the herniation and reducing risks associated with inaccurate needle placement or uncontrolled enzyme diffusion. However, the application effect of CECCN on pain scores and lumbar functional outcomes in LDH patients presents not clear.

As a result, this research aims to evaluate the effects of CECCN on pain scores and lumbar functional outcomes in LDH patients.

## Methods

### Study design

This study employed a non-randomized, comparative design. The experimental (CECCN) group consisted of 150 patients prospectively enrolled between January 2025 and December 2025. Data for this group were provided by five centers: the Department of Pain, The Second Affiliated Hospital of Guizhou Medical University (*n* = 70), the Department of Pain, Affiliated Hospital of Guizhou Medical University (*n* = 20), the Department of Pain, Affiliated Hospital of Zunyi Medical University (*n* = 20), the Department of Pain, Guiyang Fourth People’s Hospital (*n* = 20), and the Department of Rehabilitation, Tianzhu County People’s Hospital (*n* = 20). The control (ICCN) group was formed by retrospectively reviewing the medical records of patients who underwent the conventional ICCN procedure at the Department of Pain, The Second Affiliated Hospital of Guizhou Medical University between January 2023 and December 2024, met the same inclusion and exclusion criteria, and completed the predefined outcome assessments at the required follow-up time points. A total of 300 patients with single-level LDH (L3-4, L4-5, and L5-S1 segments) were included, with 150 patients in each group. The groups were compared for baseline variables (Table 1), showing no significant differences. A TREND-compatible participant flow diagram summarizing eligibility assessment, exclusions, follow-up completion, and final analysis has been provided as Supplementary Figure S2.

**Table 1:** Baseline data between the two groups.

Characteristic	ICCN Group (*n* = 150)	CECCN Group (*n* = 150)	*χ*^2^/*t*	*p*-value
Age (years), mean ± SD	42.63 ± 4.98	42.75 ± 4.72	0.214	0.830
Gender, *n* (%)			0.053	0.817
Male	80 (53.33)	78 (52.00)		
Female	70 (46.67)	72 (48.00)		
BMI (kg/m^2^), mean ± SD	24.36 ± 3.12	24.48 ± 3.05	0.337	0.736
Symptom duration (months), mean ± SD	8.42 ± 3.67	8.28 ± 3.54	0.336	0.737
LDH segment, *n* (%)			0.317	0.853
L3-4	56 (37.33)	58 (38.67)		
L4/5	60 (40.00)	62 (41.33)		
L5-S1	34 (22.67)	30 (20.00)		
Preoperative pain location, *n* (%)			0.217	0.641
Radicular pain predominant	128 (85.33)	131 (87.33)		
Back pain predominant	22 (14.67)	19 (12.67)		
Motor weakness present, *n* (%)	43 (28.67)	40 (26.67)	0.151	0.698
Smoking status, *n* (%)			0.341	0.559
Current smoker	52 (34.67)	48 (32.00)		
Non-smoker	98 (65.33)	102 (68.00)		
History of previous lumbar surgery/trauma, *n* (%)	8 (5.33)	6 (4.00)	0.302	0.583

SD, standard deviation; BMI, body mass index; LDH, lumbar disc herniation. *p*-values were calculated using independent *t*-test for continuous variables and chi-square test for categorical variables.

### Inclusion and exclusion criteria

#### Inclusion criteria

(1) Aged 18 to 70 years; (2) Single-level LDH (L3-4, L4-5, or L5-S1) confirmed by magnetic resonance imaging (MRI) before intervention, with the herniated component clearly compressing the corresponding nerve root or thecal sac; (3) Persistent radicular pain (leg pain greater than back pain), with or without neurological deficits (e.g., sensory abnormality, motor weakness, diminished reflex), for a minimum of 6 weeks; (4) Documented failure of at least 6 weeks of adequate conservative treatment before intervention, including but not limited to pharmacotherapy (NSAIDs, neuropathic pain agents), physical therapy, and/or at least one epidural steroid injection.

#### Exclusion criteria

(1) Cauda equina syndrome or severe/progressive neurological deficit requiring urgent surgical intervention; (2) Spinal stenosis (central canal stenosis or severe lateral recess stenosis) deemed the primary pain generator; (3) Spondylolisthesis (grade II or higher), spinal instability, fracture, tumor, or active infection; (4) Previous spine surgery at the index level; (5) Known allergy or contraindication to collagenase, iodinated contrast media, or local anesthetics; (6) Coagulopathy or current use of anticoagulant medication that could not be safely withheld per procedural guidelines; (7) Pregnancy or lactation; (8) Severe comorbid conditions (e.g., uncontrolled diabetes, cardiopulmonary disease) posing significant anesthesia or procedural risk; (9) Psychological disorders or ongoing litigation that could interfere with outcome assessment.

### Treatments

The ICCN procedures were performed at the Department of Pain, The Second Affiliated Hospital of Guizhou Medical University, whereas the CECCN procedures were performed across the participating centers under standardized procedural protocols by experienced interventional pain physicians using real-time image guidance. The ICCN group received conventional ICCN under fluoroscopic guidance using a digital subtraction angiography (DSA) system (TOSHIBA INFX-9000 V), for LDH at L3/4, L4/5, or L5/S1 levels. Under DSA guidance, a spinal needle was advanced via a standard posterolateral approach into the central nucleus pulposus of the target intervertebral disc. The needle trajectory passed through the “safe triangle” of Kambin, bounded by the exiting nerve root superiorly, the superior endplate of the inferior vertebra inferiorly, and the lateral border of the facet joint medially. Precise intradiscal needle placement was confirmed by lateral and anteroposterior fluoroscopic views, ensuring the needle tip was located centrally within the disc space. Prior to collagenase injection, a small volume of contrast medium was injected to perform discography, confirming the degenerative pattern of the disc and the integrity of the annulus fibrosus. Subsequently, a definitive dose of collagenase (1,200 units in 2.0 mL normal saline, Grand Life Science (Anshan) Co., Ltd.) was injected slowly into the nucleus pulposus. Post-procedure, patients were maintained in a strict prone position for 6–8 h to promote localization of the enzyme within the disc.

The CECCN group received CECCN under computed tomography (CT) guidance (Philips Brilliance CT 64 Slice) for LDH at L3/4, L4/5, or L5/S1 levels. The use of CT guidance allowed for multiplanar reconstruction (axial, coronal, and sagittal views), enabling three-dimensional confirmation of the catheter tip’s position in direct contact with or circumferentially covering the herniated material. Under CT guidance, a Tuohy needle was inserted into the sacral canal via the sacrococcygeal hiatus. A flexible, radio-opaque epidural catheter was then advanced through the needle and meticulously navigated cephalad within the anterior epidural space. Under continuous fluoroscopic control, the catheter tip was steered to achieve direct contact with or circumferential coverage of the herniated material at the target level(s). For higher levels such as L3/4, skilled catheter manipulation was required to navigate the lumbar lordotic curve. Extradural catheter positioning was confirmed by injecting a small amount of contrast agent, which demonstrated a perilesional distribution pattern enveloping the disc protrusion without intradiscal, intravascular, or intrathecal spread. The same dose of collagenase (1,200 units in 2.0 mL normal saline) was then administered slowly through the catheter, enabling broad, direct application onto the surface of the herniation within the epidural space. Following drug delivery, the catheter was removed, and standard post-injection positioning protocols were followed, consistent with the ICCN group. To improve procedural reproducibility, the key CECCN stages were standardized as follows: (1) the patient was positioned prone and the sacrococcygeal hiatus was identified under image guidance; (2) a Tuohy needle was inserted into the sacral canal, after which a flexible radio-opaque epidural catheter was introduced through the needle; (3) the catheter was advanced cephalad within the anterior epidural space to the symptomatic level, with careful steering to reach the ventral aspect of the herniated fragment; (4) a small amount of contrast agent was injected to verify perilesional extradural spread and to exclude intrathecal, intravascular, or unintended intradiscal distribution; (5) collagenase (1,200 units in 2.0 mL normal saline) was slowly delivered through the catheter to maximize contact with the surface of the herniated material; and (6) the catheter was withdrawn after drug administration and the patient underwent routine post-procedural observation. A representative step-by-step schematic of sacral access, epidural catheter navigation, contrast confirmation, and circumferential perilesional collagenase delivery has been added as Supplementary Figure S1. Because CECCN requires more complex catheter manipulation than conventional ICCN, particular attention was paid to procedural standardization to reduce operator-dependent variability.

### Outcomes

The operation time and hospital stay of both groups were recorded. All clinical outcomes were assessed according to the same predefined evaluation framework in both groups. For the retrospectively analyzed ICCN group, these data were extracted only when the corresponding outcome measures had been recorded in the medical records at the same scheduled follow-up time points used for the prospective CECCN group.

The degree of pain was evaluated using the numeric rating scale (NRS) before the intervention as well as 1 day, 7 days, 1 month, 3 months and 6 months following the intervention (11). The patients marked the corresponding position on the ruler marked from 0 to 10 on their own according to their own feelings. 0 points represented no pain, and 10 points represented unbearable severe pain.

Lumbar spine function and degree of lumbar spine disorder: The Japanese Orthopaedic Association scores (JOA) was implemented for assessing lumbar spine function before the intervention and 3 months and 6 months following the intervention (12). The lower the score, the more significant the functional impairment. The Oswestry Disability Index (ODI) was implemented for assessing lumbar spine dysfunction before the intervention and 3 months and 6 months after the intervention (13). This scale consists of 10 questions, each rated from 0 to 5 points, with higher scores representing more severe dysfunction.

Clinical efficacy at 6 months after the intervention was assessed using the modified Macnab criteria (14). For categorical efficacy assessment, clinical success was defined as an “excellent” or “good” outcome according to the modified Macnab criteria. Excellent: The original pain symptoms completely disappeared after treatment, and the individual resumed their previous work and life; Good: There were mild symptoms after treatment, with slight limitations in activity, and no need to take painkillers; Fair: The symptoms improved after treatment, with limited activity, and non-steroidal anti-inflammatory drugs were required; Poor: There was no significant difference before and after treatment, and painkillers were needed; normal activities and life were affected. The excellent and good rates of the two groups were therefore calculated as the clinical success rate. No prespecified percentage reduction in pain score was used as the formal criterion for clinical success in this study.

The quality of life was evaluated using the 36-item Short Form (SF-36) health survey before the intervention and 3 months and 6 months following the intervention (15). The SF-36 was analyzed as a continuous measure of health-related quality of life and was not used to define the binary clinical success endpoint. The SF-36 consists of 36 items, covering 8 dimensions such as physical function, mental health, and vitality, with a total score ranging from 0 to 100. The higher the score, the better the quality of life.

The occurrence of complications including infection, subcutaneous hematoma, and nerve injury in both groups within 6 months after the intervention were recorded. Complications were further classified according to type, severity (adapted from the Clavien–Dindo classification for interventional procedures), management strategies, and clinical outcomes. Any systemic allergic reactions (e.g., anaphylaxis, urticaria, bronchospasm) were also recorded.

### Statistical analysis

SPSS 20.0 was implemented for statistical analysis. Categorical data were expressed as frequency and percentage, and the *χ*^2^ test test was used for group comparisons. Measurement data that conformed to a normal distribution were presented as mean ± standard deviation, and *t*-test was used to compare the difference between the two groups. Repeated measurement analysis of variance (ANOVA) was used to compare the measurement data at multiple time points. Multivariable logistic regression analysis was conducted to evaluate the independent effect of treatment on clinical success (modified Macnab excellent/good rate at 6 months), adjusting for age, sex, disease duration, herniation level, baseline NRS and ODI scores, and disc morphology. Adjusted odds ratios with 95% confidence intervals were calculated. Statistical significance was set at *p* < 0.05.

## Results

### Participant flow and baseline data

A TREND-compatible participant flow diagram is presented in Supplementary Figure S2. In the CECCN group, 178 patients were assessed for eligibility and 28 were excluded, including 16 who did not meet the inclusion criteria, 8 who declined to participate, and 4 with contraindications; 150 patients were ultimately enrolled. In the ICCN group, 186 patients were assessed for eligibility and 36 were excluded, including 24 who did not meet the inclusion criteria and 12 with incomplete medical records; 150 patients were ultimately enrolled. All enrolled patients in both groups completed the 6-month follow-up, no patients were lost to follow-up, no outcome data were missing, and all 150 patients in each group were included in the final analysis. The groups were compared for baseline variables including age, gender, BMI, symptom duration, LDH segment, preoperative pain location, motor weakness, smoking status, and history of previous lumbar surgeries or trauma (Table 1), showing no significant differences between groups. (*p* > 0.05, Table 1).

### Operation time and hospital stay

**Figure 1:**
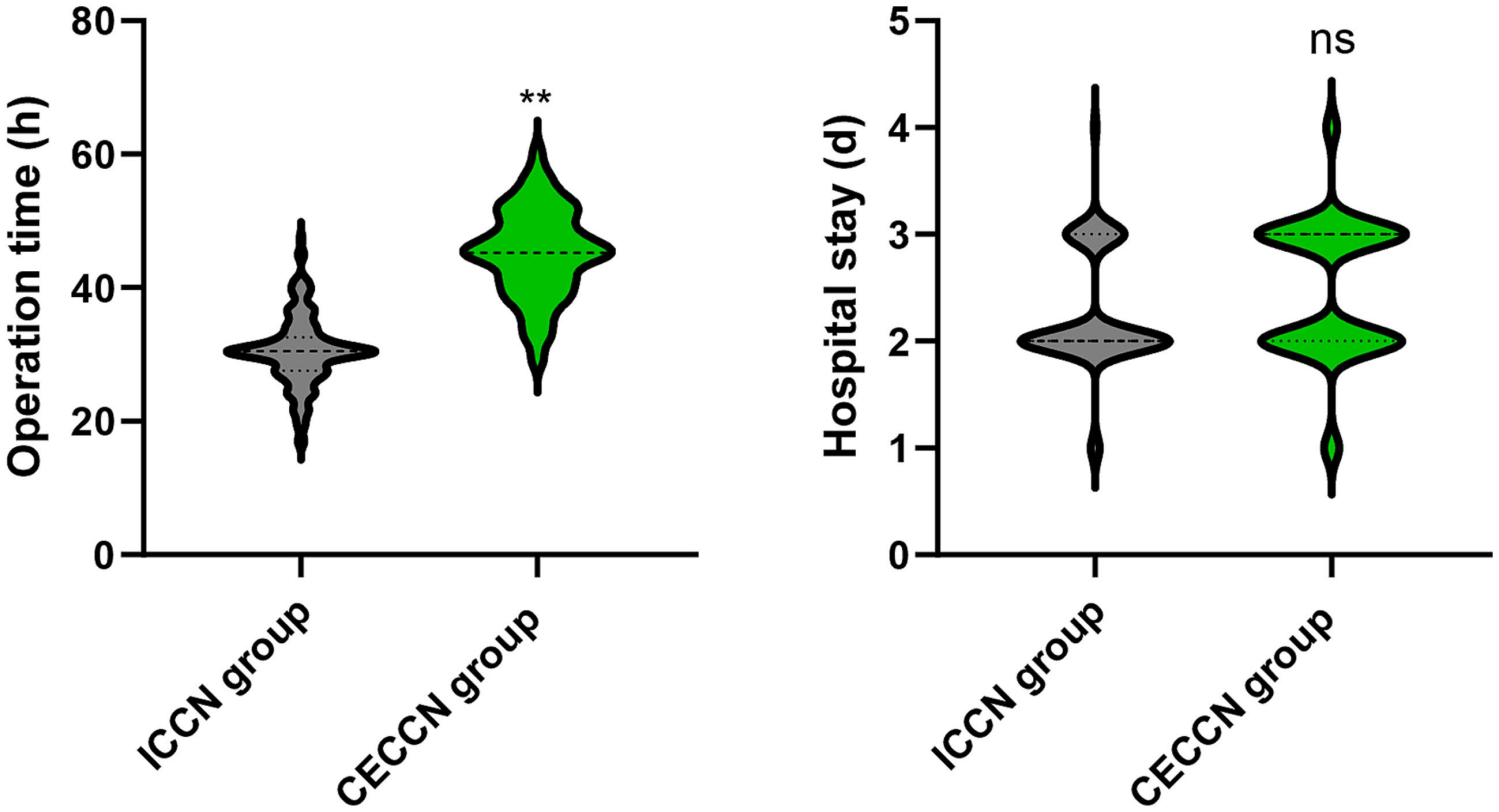
Operation time and hospital stay between the two groups. *p* < 0.01, meant the difference was not significant.

Relative to the ICCN group, the operation time of the CECCN group were longer (*p* < 0.01). No difference was seen in the hospital stay between both groups (*p* > 0.05, Figure 1).

### Degree of pain

**Figure 2:**
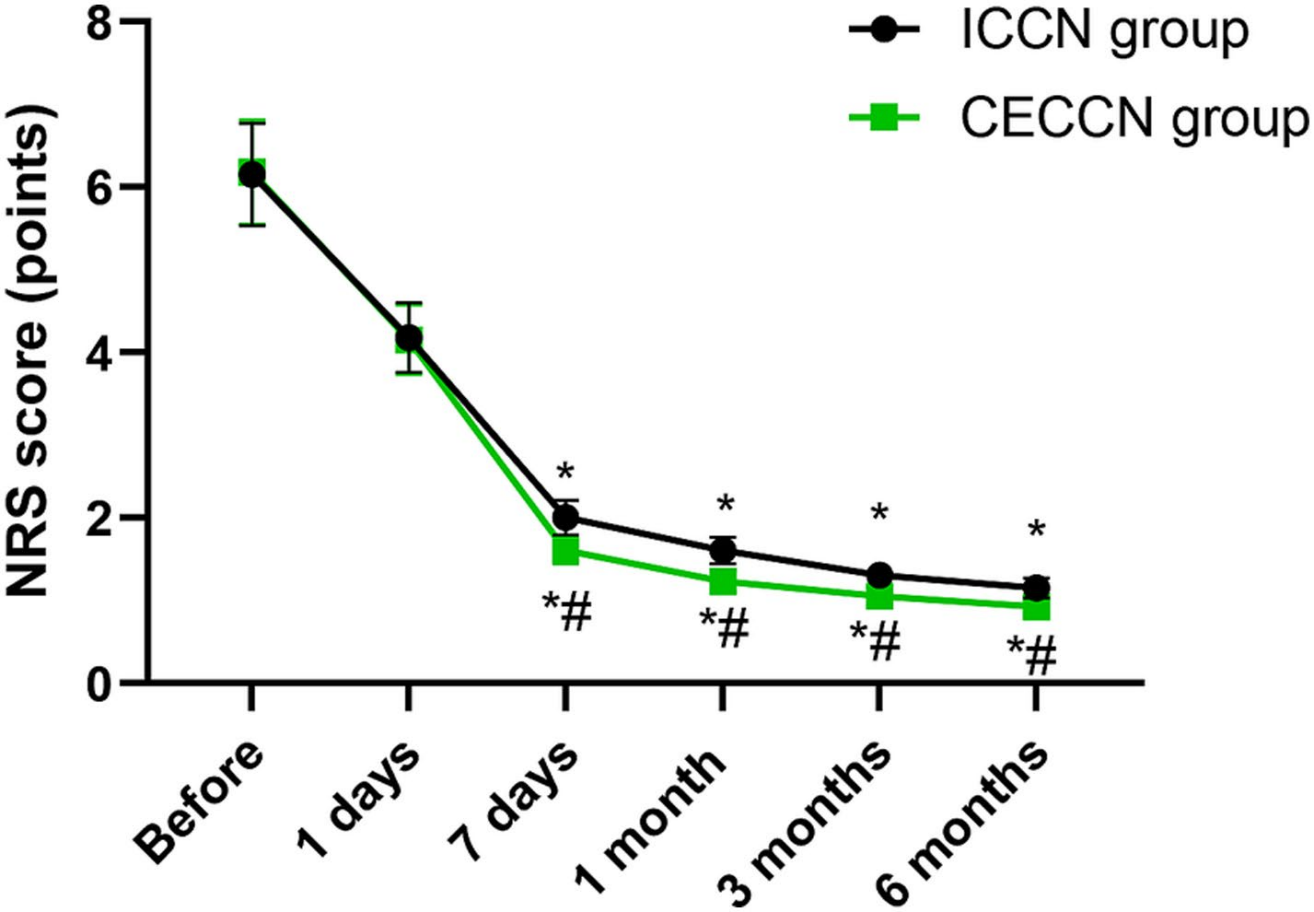
Degree of pain between the two groups at different time points. *p < 0.05, compared with before intervention; #*p* < 0.05, compared with ICCN group.

Before intervention and 1 day after intervention, no differences were seen in NRS scores between both groups (p > 0.05). Compared with before intervention, the NRS scores of both groups decreased at 7 days, 1 month, 3 months as well as 6 months after intervention (*p* < 0.05), and the NRS scores of the CECCN group at 7 days, 1 month, 3 months as well as 6 months following intervention were all lower than those of the ICCN group (*p* < 0.05, Figure 2).

### Lumbar spine function and degree of lumbar spine disorder

**Figure 3:**
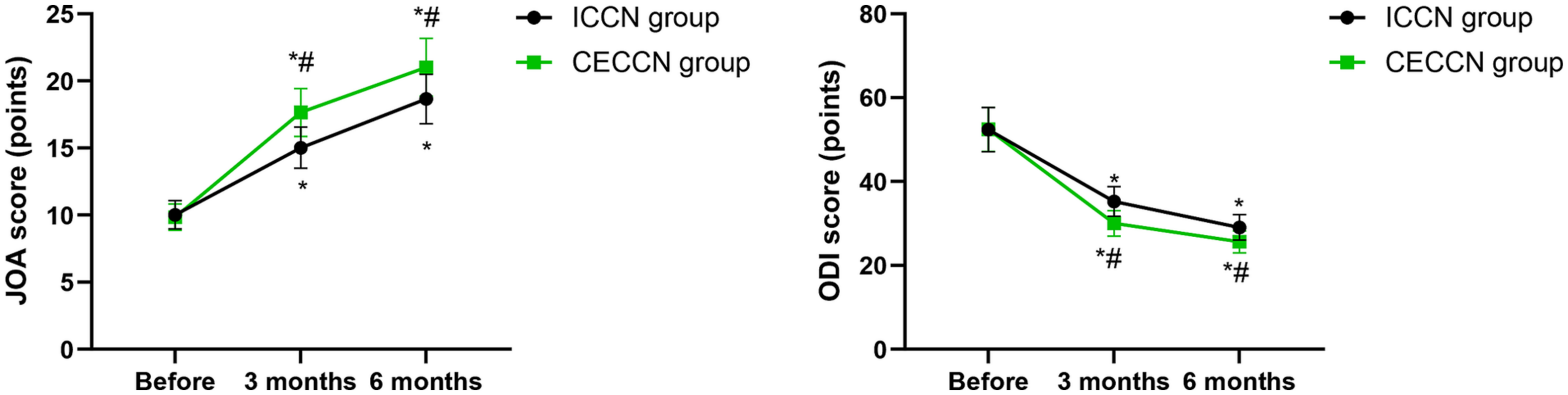
Lumbar spine function and degree of lumbar spine disorder between the two groups at different time points. *p < 0.05, compared with before intervention; #*p* < 0.05, compared with ICCN group.

Before intervention, no differences were seen in JOA and ODI scores between the two groups (*p* > 0.05). Compared with before intervention, the JOA scores of both groups increased at 3 months and 6 months following intervention (*p* < 0.05), while the ODI scores of both groups decreased at 3 months and 6 months following intervention (*p* < 0.05). At 3 months and 6 months following intervention, the JOA scores of the CECCN group were higher than those of the ICCN group (p < 0.05), and the ODI scores of the CECCN group were lower than those of the ICCN group (*p* < 0.05, Figure 3).

### Clinical efficacy

Compared with the ICCN group, the excellent and good rate of the CECCN group presented higher (*p* < 0.05, Table 2).

**Table 2:** Clinical efficacy between the two groups.

Groups	Cases	Excellent	Good	Fair	Poor	Excellent and good rate
ICCN group	150	97 (64.67)	30 (20.00)	12 (8.00)	11 (7.33)	127
CECCN group	150	111 (74.00)	31 (20.67)	6 (4.00)	2 (1.33)	142
*χ* ^2^						8.094
*p*						0.004

### Quality of life

**Figure 4:**
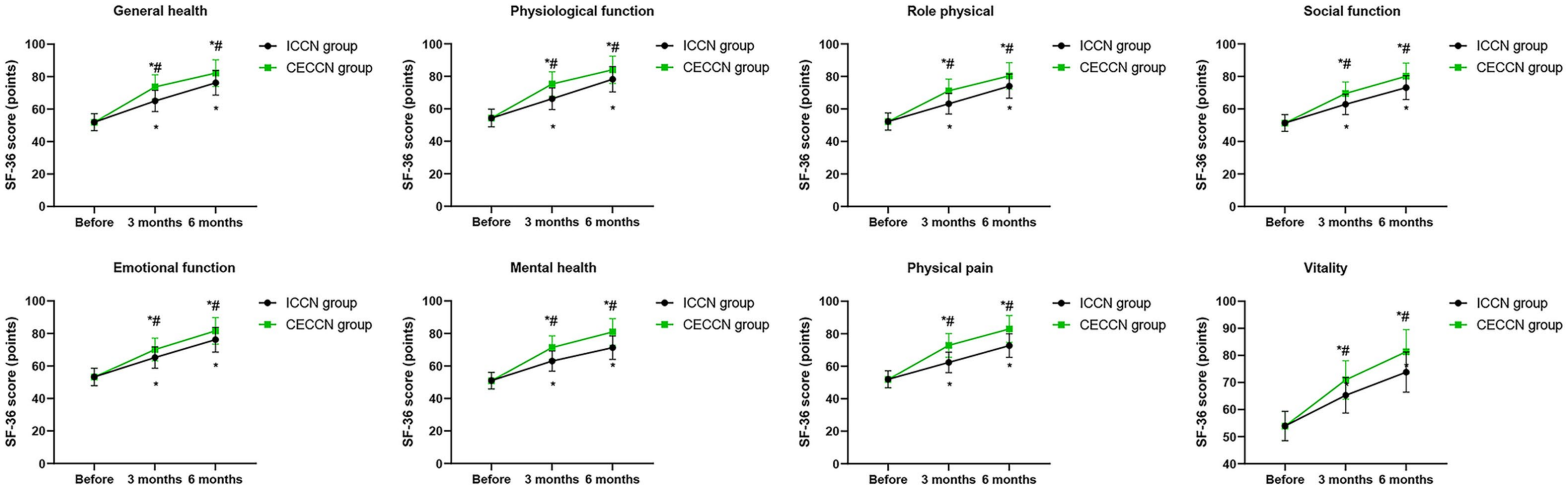
Quality of life between the two groups at different time points. *p < 0.05, compared with before intervention; #*p* < 0.05, compared with ICCN group.

Before intervention, no differences were seen in SF-36 scores between both groups (*p* > 0.05). Compared with before intervention, the SF-36 scores of both groups increased at 3 months and 6 months following intervention (p < 0.05), and the SF-36 scores of the CECCN group at 3 months and 6 months following intervention were all higher than those of the ICCN group (p < 0.05, Figure 4).

### Occurrence of complications

Compared with the ICCN group, the occurrence of complications in the CECCN group was significantly lower (4.66% vs. 11.34%, *p* = 0.033). Detailed information regarding complication type, severity, management, and outcomes is presented in Table 3. All complications in both groups were mild to moderate (Grade I-II) and resolved with conservative management or minor interventions; no Grade III or higher complications occurred. The most common complication was subcutaneous hematoma, which resolved spontaneously with observation. Infections were successfully treated with oral or intravenous antibiotics, and all nerve injuries (transient sensory or motor deficits) resolved completely within 1–3 months. Importantly, no cases of anaphylaxis or systemic allergic reactions were observed in either group.

**Table 3:** Occurrence of complications between the two groups.

Complication type	ICCN group (*n* = 150)	CECCN group (*n* = 150)	Severity grade*	Management	Outcome
Infection	7 (4.67%)	2 (1.33%)			
Superficial surgical site infection	5	2	Grade II	Oral antibiotics (7–10 days)	Resolved without sequelae
Deep infection/diskitis	2	0	Grade II	IV antibiotics (2–3 weeks)	Resolved without sequelae
Subcutaneous hematoma	6 (4.00%)	3 (2.00%)	Grade I	Observation, ice packs	Spontaneous resolution within 1–2 weeks
Nerve injury	4 (2.67%)	2 (1.33%)			
Transient sensory deficit	3	2	Grade I	Observation, neurotropic vitamins	Complete recovery within 1–3 months
Transient motor weakness	1	0	Grade II	Observation, physical therapy	Complete recovery within 3 months
Anaphylaxis/systemic allergic reaction	0 (0%)	0 (0%)	–	–	–
Total complications	17 (11.34%)	7 (4.66%)			
*p*-value		0.033			

Severity grading adapted from Clavien–Dindo classification for interventional procedures: Grade I, mild, requiring no or minimal intervention (observation, ice packs); Grade II, moderate, requiring pharmacological treatment (antibiotics) or minor intervention; Grade III, severe, requiring surgical or radiological intervention; Grade IV, life-threatening; Grade V, death.

### Subgroup analysis based on disc morphology, age, and comorbidities

To explore whether disc morphology influenced treatment response, patients were stratified into three subgroups according to preoperative MRI findings: contained, extruded, and sequestrated herniation. The distribution of morphological types was comparable between the two groups (Table 4). At 6-month follow-up, the modified Macnab excellent/good rate was analyzed within each subgroup. In patients with contained herniation, no significant difference was observed between the ICCN and CECCN groups (85.0% vs. 90.2%, respectively; *p* = 0.25). For extruded herniations, the CECCN group showed a significantly higher success rate compared to the ICCN group (95.0% vs. 80.0%; *p* = 0.04). The most pronounced difference was found in the sequestrated subgroup: the excellent/good rate was 50.0% in the ICCN group versus 100.0% in the CECCN group (*p* = 0.02).

**Table 4:** Subgroup analysis of clinical efficacy at 6 months based on disc morphology.

Subgroup	ICCN group (*n* = 150)	CECCN group (*n* = 150)	*χ* ^2^	*p*-value
Disc morphology				
Contained	85/100 (85.0%)	92/102 (90.2%)	1.32	0.25
Extruded	32/40 (80.0%)	38/40 (95.0%)	4.11	0.04
Sequestrated	5/10 (50.0%)	8/8 (100.0%)	5.23	0.02
Age group				
< 40 years	32/35 (91.4%)	33/34 (97.1%)	1.03	0.31
40–60 years	79/95 (83.2%)	91/96 (94.8%)	6.58	0.01
> 60 years	16/20 (80.0%)	18/20 (90.0%)	4.43	0.04
Comorbidity status				
No comorbidities	95/110 (86.4%)	102/108 (94.4%)	3.98	0.046
Hypertension only	24/30 (80.0%)	28/30 (93.3%)	2.31	0.13
Diabetes mellitus*	8/12 (66.7%)	12/12 (100%)	4.80	0.03

Data are presented as number of patients with excellent/good outcome / total patients in subgroup (%). *p*-values were calculated using the chi-square test. *Diabetes mellitus group includes patients with or without hypertension.

CECCN consistently yielded higher excellent/good rates across all age groups. The difference was statistically significant in the 40–60 years (94.8% vs. 83.2%, *p* = 0.01) and >60 years (90.0% vs. 80.0%, p = 0.04) cohorts. In patients younger than 40 years, the success rate was high with both techniques, and the difference did not reach significance (*p* = 0.31), possibly due to the smaller sample size and generally favorable prognosis in younger individuals.

In patients without comorbidities, CECCN showed a modest but significant advantage over ICCN (94.4% vs. 86.4%, *p* = 0.046). Among patients with hypertension alone, the success rate favored CECCN (93.3% vs. 80.0%), but the difference was not statistically significant (*p* = 0.13), likely due to the limited sample size. Most notably, in patients with diabetes mellitus, CECCN achieved 100% excellent/good outcomes compared to 66.7% in the ICCN group (*p* = 0.03), suggesting that targeted extradural enzyme delivery may overcome the impaired healing response associated with diabetes.

### Multivariable logistic regression analysis

To further evaluate the independent effect of treatment after adjusting for potential confounders, multivariable logistic regression analysis was performed. As shown in Table 5, CECCN remained independently associated with significantly higher odds of achieving clinical success at 6 months (adjusted OR = 3.24; 95% CI: 1.58–6.64; *p* = 0.001), after controlling for age, sex, disease duration, herniation level, baseline NRS, baseline ODI, and disc morphology. Among the covariates, non-contained disc morphology showed a trend toward lower odds of success (adjusted OR = 0.51; *p* = 0.05).

**Table 5:** Multivariable logistic regression analysis of factors associated with clinical success (modified macnab excellent/good at 6 months).

Variable	Category	Adjusted OR	95% CI	*p*-value
Treatment group	CECCN vs. ICCN	3.24	1.58–6.64	0.001
Age	Per 1-year increase	0.98	0.95–1.01	0.18
Sex	Male vs. Female	1.12	0.63–1.99	0.70
Disease duration	Per 1-year increase	0.94	0.81–1.09	0.42
Herniation level	L4/5 vs. L3/4	0.87	0.41–1.85	0.72
	L5/S1 vs. L3/4	0.79	0.34–1.83	0.58
Baseline NRS	Per 1-point increase	0.92	0.79–1.07	0.28
Baseline ODI	Per 1% increase	0.96	0.93–1.00	0.06
Disc morphology	Non-contained vs. contained	0.51	0.26–1.00	0.05

OR, odds ratio; CI, confidence interval; NRS, Numeric Rating Scale; ODI, Oswestry Disability Index. Non-contained includes extruded and sequestrated types.

## Discussion

This research demonstrates that CECCN is a more effective and potentially safer minimally invasive intervention for single-level LDH refractory to conservative care than the conventional intradiscal injection technique. The CECCN group achieved superior outcomes across multiple validated metrics—including pain relief (NRS), functional recovery (JOA, ODI), clinical success rate (modified Macnab), and quality of life (SF-36)-at 3 and 6 months follow-up. While the novel technique required a longer operative time, it was associated with a significantly lower incidence of complications.

The primary strength of the catheter-based extradural technique lies in its fundamental pharmacokinetic advantage: direct, targeted drug delivery. By navigating a catheter to the anterior epidural space and delivering collagenase circumferentially around the herniated fragment, this method ensures maximal enzyme-to-substrate contact. This directly addresses the well-documented limitations of the intradiscal approach, where enzyme distribution within the central nucleus is unpredictable, diffusion to the actual site of nerve compression (the extruded fragment) is inefficient, and a significant proportion of the drug may be pharmacologically “wasted” within the disc matrix. Our finding of significantly lower NRS scores in the CECCN group as early as 7 days post-procedure strongly supports the premise of more rapid and effective dissolution of the compressive pathology. This aligns with biomechanical studies suggesting that direct epidural application leads to faster degradation of herniated tissue compared to the indirect intradiscal route (16). Prior discographic and CT-discographic studies have shown that injectate behavior after intradiscal administration is highly dependent on disc structure. In normal discs, contrast tends to remain confined to the nucleus pulposus, whereas in degenerated discs it may extend along annular fissures; in extruded lesions, annular disruption with epidural contrast extravasation may occur, indicating that intradiscal injection does not necessarily ensure uniform exposure of the extruded fragment to the injected agent (17). By contrast, imaging studies of epidural injection have demonstrated that epidural injectate can spread longitudinally and circumferentially around the target level, including toward the anterior epidural space, and that the final distribution pattern is influenced by procedural and physicochemical factors such as injectate volume and viscosity (18). These comparative distribution characteristics provide additional mechanistic support for the pharmacokinetic advantage of CECCN. Rather than relying on diffusion from the nucleus through a structurally disrupted disc, catheter-based perilesional epidural delivery may achieve more direct and consistent enzyme contact with the surface of the herniated material (17).

The superior functional outcomes (JOA, ODI) and higher excellent/good rates (Macnab) at medium-term follow-up can be attributed to this efficient decompression and a potentially more favorable biological response. The intradiscal technique, by requiring annular puncture and delivering collagenase primarily within the disc space, may promote additional local inflammatory activation within the injured disc environment and does not directly target the epidural inflammatory milieu surrounding the herniated fragment (19). In contrast, the epidural application may allow for more focused dissolution of the offending fragment while minimizing collateral damage to the intact disc architecture, leading to more robust and durable functional recovery. This hypothesis is further corroborated by our quality of life (SF-36) results, which showed greater improvement in the CECCN group.

The longer operative time for the catheter-based technique was an expected trade-off, reflecting the procedural complexity of sacral access, epidural navigation, and precise catheter tip positioning under fluoroscopy. Crucially, this did not translate into a longer hospital stay, indicating that the added procedural time did not increase acute peri-procedural morbidity or alter discharge criteria.

Perhaps the most clinically significant finding was the reduced complication rate in the CECCN group. The conventional transforaminal intradiscal approach carries inherent, albeit low, risks associated with needle traversal of the “safe triangle,” including direct nerve root injury, vascular puncture, and unintentional intrathecal injection (20). Our catheter-based technique, utilizing a caudal epidural entry far from the neural elements at the pathological level, inherently avoids these needle-related risks during initial access. Furthermore, the controlled, low-pressure injection via a catheter positioned against the herniation minimizes the risk of inadvertent enzyme diffusion into vulnerable structures (e.g., thecal sac, epidural veins), a potential source of neurological complications or reduced efficacy seen with high-pressure bolus injections. The lower complication profile enhances the safety proposition of chemical dissolution as a true alternative to surgery. Importantly, although collagenase-based chemonucleolysis carries a theoretical risk of hypersensitivity, no anaphylaxis or other systemic allergic reaction was observed in either group in the present study. A detailed analysis of the complications observed in this study provides further support for the safety advantage of CECCN. All complications in both groups were mild to moderate (Grade I-II) and resolved with conservative management or minor interventions; no Grade III or higher complications occurred. The most common complication was subcutaneous hematoma, which resolved spontaneously with observation. Infections were successfully treated with oral or intravenous antibiotics, and all nerve injuries (transient sensory or motor deficits) resolved completely within 1–3 months. Notably, the CECCN group not only had a significantly lower overall complication rate but also demonstrated a trend toward milder severity, with no deep infections and no transient motor weakness compared to the ICCN group.

Furthermore, a significant methodological distinction between the groups was the image guidance modality: DSA for ICCN versus CT for CECCN. While DSA provides excellent real-time two-dimensional fluoroscopic visualization for needle placement within the disc, CT guidance offers superior three-dimensional anatomical resolution. The use of CT in the CECCN group was integral to the technique’s precision, allowing definitive multiplanar verification of catheter-to-herniation contact. This difference in guidance technology is a potential confounding factor and may itself have contributed to the observed outcome differences by ensuring more accurate drug delivery in the experimental arm. It represents both a technical advancement and a study limitation when comparing the procedural techniques directly.

The subgroup analysis based on disc morphology provides additional insight into the differential efficacy of the two chemonucleolysis techniques. Recent studies of intradiscal condoliase have shown that treatment response is influenced by pretreatment MRI and herniation-related characteristics, including herniation occupancy, Pfirrmann grade, disc height, and herniated mass size, highlighting the clinical importance of morphology-based patient selection (21–23). In our cohort, both techniques performed well for contained herniations, whereas the between-group difference became more apparent in the extruded and sequestrated subgroups. This pattern is biologically plausible because the effectiveness of conventional intradiscal chemonucleolysis depends not only on enzyme activity itself, but also on how successfully the injected agent reaches the herniated target tissue through the disc environment (22, 23). In particular, the inferior ICCN outcome in the sequestrated subgroup and the uniformly favorable CECCN outcome in the same subgroup suggest that direct extradural delivery may improve enzyme-to-target contact when the herniated fragment is anatomically separated from the parent disc. This interpretation should be considered hypothesis-supporting rather than definitive, given the limited number of sequestrated cases, but it is consistent with the broader contemporary view that careful anatomical and imaging-based selection is critical for optimizing chemonucleolysis outcomes (9, 21). Therefore, our findings further support the value of preoperative MRI-based morphological classification not only for baseline characterization but also for anticipating which patients are more likely to benefit from different drug-delivery strategies.

Several limitations of this study warrant mention. First, the follow-up period of 6 months, while sufficient to demonstrate short- to medium-term efficacy and safety, is not long enough to assess long-term recurrence, durability of symptom relief, or delayed complications. At present, mature follow-up data beyond 6 months are not available for the current cohort. Therefore, the theoretical advantage of the epidural technique in bypassing the annular defect—a potential pathway for recurrence after intradiscal therapy—requires confirmation in studies with extended follow-up. Second, this was a non-randomized comparative study, which inherently introduces the possibility of selection bias and residual confounding. To reduce this risk, we applied the same eligibility criteria across both groups, used the same predefined outcome assessment framework, confirmed baseline comparability using an expanded set of demographic and clinical variables, and further performed multivariable adjustment for potential confounders. Nevertheless, the multicenter prospective design of the CECCN group and the single-center retrospective design of the ICCN group, as well as the different enrollment periods, may still have introduced center-related and temporal confounding. Although measured baseline variables such as age, sex, BMI, symptom duration, LDH segment, motor weakness, smoking status, and relevant lumbar history were well balanced between groups, unmeasured factors may still have influenced the observed treatment effect. Third, the difference in image-guidance modality (DSA for ICCN versus CT for CECCN), although clinically appropriate for the technical requirements of each procedure, remains a potential source of confounding that may have affected procedural precision or outcomes independently of the drug-delivery route itself. Future studies using the same guidance modality in both groups would help isolate the specific contribution of catheter-based epidural delivery. Fourth, although both procedures were performed by skilled interventionalists using standardized protocols, operator experience was not analyzed as an independent variable, and this was not a formal operator-matched study. Therefore, a learning-curve effect, particularly for the more technically demanding CECCN procedure, cannot be completely excluded. Finally, cost-effectiveness was not evaluated. The longer procedural time and the potential additional cost associated with specialized catheters should be weighed against the observed improvements in efficacy and safety in future health-economic analyses.

## Conclusion

This study suggests that CECCN may be superior to the conventional ICCN for treating symptomatic lumbar disc herniation. By enabling precise, direct application of the enzyme to the pathological target, it yields faster and greater pain relief, better functional recovery, higher patient satisfaction, and a safer procedural profile. This technique represents a meaningful evolution in percutaneous disc decompression, offering a potent minimally invasive option for patients seeking an alternative between conservative therapy and open surgery. Future research should focus on long-term outcomes, technical standardization, the independent contribution of advanced imaging guidance, and cost–benefit analyses.

## Data Availability

The original contributions presented in the study are included in the article/Supplementary material, further inquiries can be directed to the corresponding author.

## Supplementary material

Supplementary Figure S1 Representative schematic of the CECCN procedure. **(A–D)** illustrate the major steps of CECCN: **(A)** sacral access through the sacrococcygeal hiatus; **(B)** cephalad advancement of the epidural catheter toward the target lumbar level; **(C)** contrast confirmation of extradural perilesional spread around the herniated fragment; and **(D)** circumferential collagenase delivery onto the surface of the herniation. CECCN, catheter-based extradural collagenase chemonucleolysis.

Supplementary Figure S2 Participant flow diagram. The diagram summarizes patient flow in this non-randomized comparative study, including eligibility assessment, exclusion with reasons, enrollment into the CECCN and ICCN groups, completion of 6-month follow-up, missing data, and final analysis.
