# Supplementary figures and images for: Effects of catheter-based extradural collagenase chemonucleolysis on pain scores and lumbar function in patients with lumbar disc herniation
Source: Front Med (Lausanne). 2026 Apr 13;13:1807178. doi: 10.3389/fmed.2026.1807178 (PMC13110996; doi:10.3389/fmed.2026.1807178)

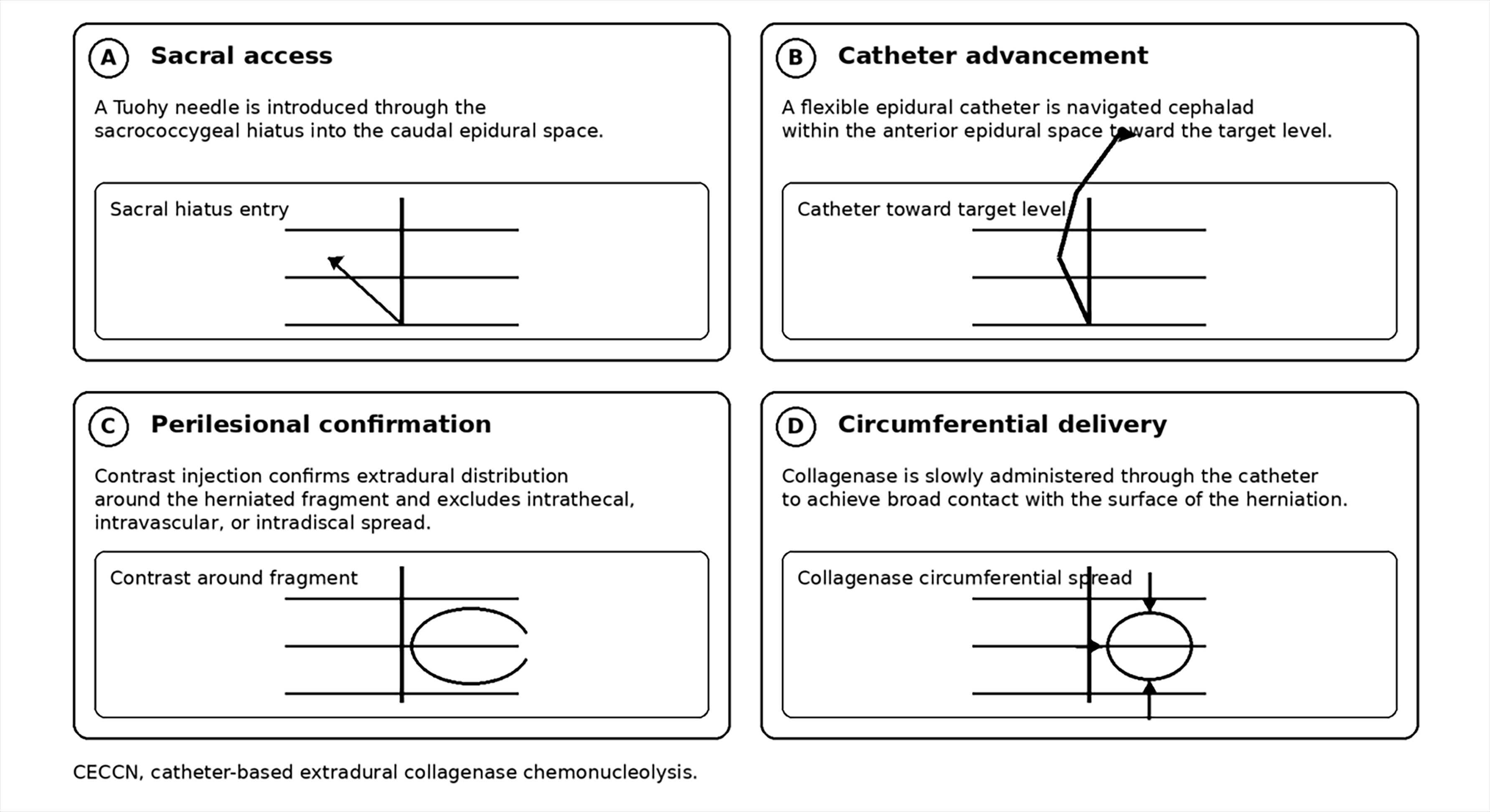

Supplement: Supplementary Figure S1 — Representative schematic of the CECCN procedure. (A–D) illustrate the major steps of CECCN: (A) sacral access through the sacrococcygeal hiatus; (B) cephalad advancement of the epidural catheter toward the target lumbar level; (C) contrast confirmation of extradural perilesional spread around the herniated fragment; and (D) circumferential collagenase delivery onto the surface of the herniation. CECCN, catheter-based extradural collagenase chemonucleolysis. [file Image_1.PNG]

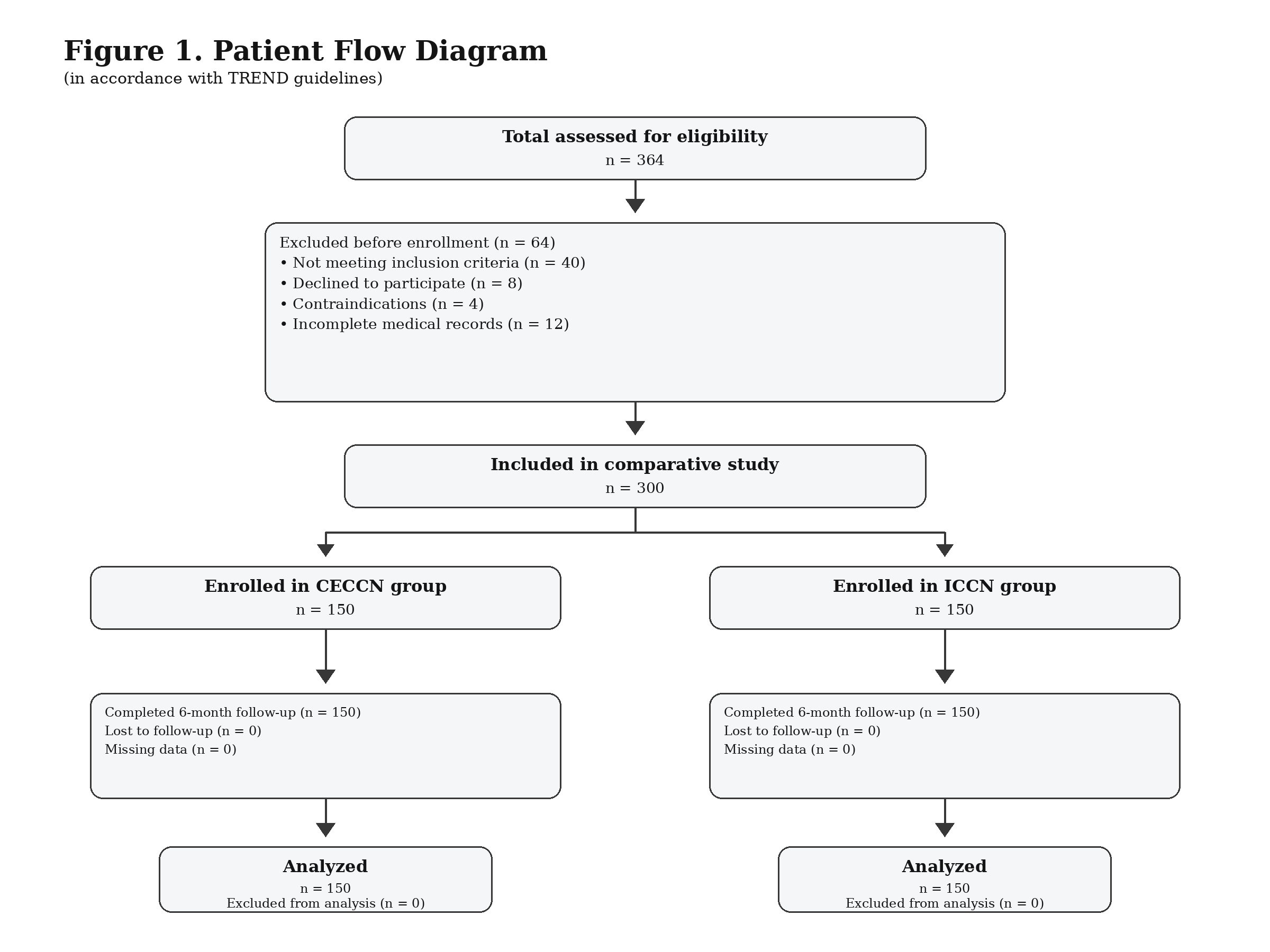

Supplement: Supplementary Figure S2 — Participant flow diagram. The diagram summarizes patient flow in this non-randomized comparative study, including eligibility assessment, exclusion with reasons, enrollment into the CECCN and ICCN groups, completion of 6-month follow-up, missing data, and final analysis. [file Image_2.PNG]
